# Compact and evenly distributed *k*-mer binning for genomic sequences

**DOI:** 10.1093/bioinformatics/btab156

**Published:** 2021-03-08

**Authors:** Johan Nyström-Persson, Gabriel Keeble-Gagnère, Niamat Zawad

**Affiliations:** JNP Solutions, Yokoami, Sumida-ku, Tokyo 130-0015, Japan; Department of R&D, Lifematics Inc., Kanda Jinbocho, Chiyoda-ku, Tokyo 101-0051, Japan; Department of Agriculture Victoria Research, AgriBio, Centre for AgriBioscience, Bundoora, VIC 3083, Australia; Department of R&D, Lifematics Inc., Kanda Jinbocho, Chiyoda-ku, Tokyo 101-0051, Japan

## Abstract

**Motivation:**

The processing of *k*-mers (subsequences of length *k*) is at the foundation of many sequence processing algorithms in bioinformatics, including *k*-mer counting for genome size estimation, genome assembly, and taxonomic classification for metagenomics. Minimizers—ordered *m*-mers where *m *<* k*—are often used to group *k*-mers into bins as a first step in such processing. However, minimizers are known to generate bins of very different sizes, which can pose challenges for distributed and parallel processing, as well as generally increase memory requirements. Furthermore, although various minimizer orderings have been proposed, their practical value for improving tool efficiency has not yet been fully explored.

**Results:**

We present Discount, a distributed *k*-mer counting tool based on Apache Spark, which we use to investigate the behaviour of various minimizer orderings in practice when applied to metagenomics data. Using this tool, we then introduce the universal frequency ordering, a new combination of frequency-sampled minimizers and universal *k*-mer hitting sets, which yields both evenly distributed binning and small bin sizes. We show that this ordering allows Discount to perform distributed *k*-mer counting on a large dataset in as little as 1/8 of the memory of comparable approaches, making it the most efficient out-of-core distributed *k*-mer counting method available.

**Availability and implementation:**

Discount is GPL licensed and available at https://github.com/jtnystrom/discount. The data underlying this article are available in the article and in its online supplementary material.

**Supplementary information:**

Supplementary data are available at *Bioinformatics* online.

## 1 Introduction

The analysis of *k*-mers, short sequence fragments of a fixed length *k*, is foundational to many methods and algorithms in bioinformatics, including genome assembly (Koren *et al.*, 2017), *k*-mer counting (Erbert *et al.*, 2017; Kokot *et al.*, 2017; Rizk *et al.*, 2013), variant calling (Audano *et al.*, 2018) and metagenomic classification (Wood and Salzberg, 2014). Due to the proliferation of next-generation sequencing (NGS) data and other types of omics data, such *k*-mer data analysis needs are constantly increasing. This has led to the need for ever more efficient algorithms and methods in this area. The *k*-mer analysis of large datasets is often computationally challenging. For example, when *k *=* *55, for the usual DNA alphabet {A, C, G, T} there exists a total of 4^55^ (approximately $1.3\times 10^{33}$) possible such *k*-mers. This large data space, usually much too large to represent in memory or on disk in its entirety, is a major source of the complexity of *k*-mer analysis. One commonly used strategy for overcoming this complexity is *k*-mer binning. Since only a small fraction of all possible *k*-mers are seen in practice for a given dataset, one aims to subdivide the data that is actually encountered into reasonably sized parts and base data processing (such as counting, manipulation, lookup of associated data) on these.

Binning is often done by grouping *k*-mers according to their *minimizers*, a technique first introduced in biological applications by Roberts *et al.* (2004). Minimizers are obtained by ordering all *m*-mers *M_i_* for some fixed *m*, where *m *<* k*, in some way: $M_{0}<M_{1}<\ldots <M_{n}$. We say that *M_i_* is smaller than *M_j_* if *i *<* j*. Each *k*-mer is then classified according to the smallest minimizer in it. Often, consecutive *k*-mers in a longer sequence will share the same *m*-minimizer. Thus, an input sequence may be split into *super-mers*, maximally long substrings where all *k*-mers share the same minimizer. This allows for the partitioning of super-mers into bins, where each bin corresponds to a minimizer, which effectively yields a hash table for *k*-mers. Grouping *k*-mers together in this way can also provide a compact representation, since super-mers are much more space efficient than representing each *k*-mer by itself.

Various minimizer orderings exist. Although simple to implement and reason about, the basic lexicographic ordering proposed by Roberts leads to very unbalanced bins in practice. This can lead to higher memory usage and to a slowdown in general, since algorithms on larger bins can be more expensive to run.

The *k*-mer counter KMC2 (Deorowicz *et al.*, 2015) introduced *minimizer signatures*, which order *m*-mers lexicographically, except that in order to reduce data skew, *m*-mers starting with AAA or ACA are given lower priority, and *m*-mers containing AA anywhere are also avoided, except for AA at the start. This helps spread out the *k*-mers somewhat and avoid certain very unbalanced bins. This ordering is also used by Gerbil (Erbert *et al.*, 2017), and by FastKmer (Ferraro Petrillo *et al.*, 2019) in a modified form with some additional rules.

The *frequency-counted* ordering was first introduced by Chikhi *et al.* (2014) for the purpose of efficient de Bruijn Graph representation. A similar approach (weighted minimizers) was also used by Jain *et al.* (2020) for long read mapping. In this ordering, rare minimizers, based on occurrence in the actual dataset in each case, are given higher priority than common minimizers.

Finally, the concept of *compact universal k-mer hitting sets* was recently introduced by Orenstein *et al.* (2016, 2017). For any given sequence of length *k* to be hit by (include) at least one sequence of length *m* in some set of *m*-mers, it is not necessary to include every *m*-mer in the set. Small sets that hit every *k*-length sequence can be precomputed. Such a set can be turned into an ordering by giving all *m*-mers not in the set lower priority, effectively excluding them. Although generating optimal universal sets is an NP-hard problem, the recently introduced PASHA (Ekim *et al.*, 2020) algorithm is able to generate near-optimal sets relatively quickly.

Although many different orderings with diverse characteristics have been proposed, it is still not clear which ordering should be preferred in practice, and many recent innovations have not yet been fully explored. Thus, to help identify the best methods for large scale omics data analysis, a practical evaluation of the various possible orderings when applied to demanding tasks is needed.

When evaluating minimizer orderings with the aim of improving software efficiency, the following measurements are useful to consider.

- **Maximum bin size.** This directly impacts performance. For tools that perform out-of-core sequential processing of bins, often the minimum memory requirement is that each bin should be able to fit in memory in its entirety. Moreover, larger bins may increase the total runtime due to the superlinear cost of algorithms such as sorting, as well as the amount of temporary data structures being allocated.
- **Flatness of distribution.** This can be measured in various ways. Since the impact of outsized large bins is much more significant than that of small bins, we focus on the proportion of *k*-mers stored in the largest 0.5% of bins. We also give the max/mean ratio of bin sizes.
- **Length of super-mers/compactness.** Longer super-mers give a more compact representation in memory and on disk, or for network transmission in a distributed setting. Equivalently, one may measure the average distance between minimizers (the inverse of their *density*), or the total number of super-mers (inversely proportional to their average length for a given dataset) (More precisely, the total number of *k*-mers represented by *n* super-mers of length *L* (in letters) is n(L−(k−1)), since each super-mer has to overlap another by (k−1) letters. Thus, the larger *L*, the smaller the fraction of pure overlap data (k−1) in the super-mer, and the more efficient the storage.).
- **Number of bins.** Many tools currently in use also try to limit the number of bins. For example, the KMC2 authors argue that one goal of a good minimizer ordering should be that ‘the number of bins should be neither too large nor too small’ (Deorowicz *et al.*, 2015). However, while limiting the number of bins is reasonable when each bin is stored in a separate file, alternative system designs allow a large number of bins to be stored together in a small number of files. Furthermore, a larger number of fine grained bins has advantages in subsequent processing. Many algorithms, such as sorting, have a lower per-item cost when applied to smaller lists of items, e.g. quicksort has a best case runtime of O(n log n) and a worst case of $O(n^{2})$. Thus, in addition to the goals stated above, we believe that the ability to generate a large number of small (but evenly distributed) bins is a desirable goal, especially if this can also be done while keeping super-mers long.

Marçais *et al.* (2017) provided a systematic study of the minimizer behaviour in practice of various widely used tools. They compared existing tools with universal sets generated by DOCKS (Orenstein *et al.*, 2016) for the parameters *m *=* *7, *k *=* *11, for a synthetic dataset as well as for the human genome. They reported the average bin size, the max/mean ratio for bins, and the mean distance between minimizers. The number of bins studied was in each case ≤16,384.

Various minimizer orderings were evaluated by Erbert *et al.* (2017), in the context of the F. vesca genome for *m *=* *6, *k *=* *28. In addition to the KMC2/signature ordering, lexicographic ordering, randomized ordering and the CGAT (lexicographic, but with C < G < A < T) ordering, they also studied *distance from pivot* (dfp), which is a modified version of the frequency-counted ordering that attempts to avoid very small bins. For this comparison, they only reported the maximum bin size and the total number of super-mers. Although compactness was measured, measurements such as the bin size distribution, or the number of bins generated in each case, were not reported.

In omics data analysis, metagenomics data has much higher complexity compared to single-species omics data, owing to the large number of distinct *k*-mers, and hence represents a challenging use case. Here, we systematically study, using two metagenomics datasets, the use of six different minimizer orderings to generate a large number of *k*-mer bins. We consider cases in the order of 10^5^ to 10^6^ bins. As far as we know, this is the first time that these minimizer orderings have been comparatively evaluated for a large number of bins, or with metagenomics data. As part of our study we propose a new ordering, *the universal frequency ordering*, which yields a very even distribution and long super-mers.

With the increasing size and complexity of omics datasets, and the high resource requirements of some omics tools, there is an increasing need for distributed algorithms (Ferraro Petrillo *et al.*, 2017). For distributed processing, being able to subdivide the workload evenly is even more important than for single-machine processing, since communication and synchronization costs, such as shuffling over the network, can be significant. One of the more popular and widely accepted frameworks for distributed data processing in recent years is Apache Spark (The Apache Software Foundation, 2020) (Spark for short), which brings a general programming model to the Hadoop distributed filesystem.

As a case study for the performance benefits of minimizer orderings, we are particularly interested in the problem of *k*-mer counting (Manekar and Sathe, 2018). In itself, this method can be used for purposes such as abundance filtering and genome size estimation, and it can also be a necessary foundation for more complex methods, such as de Bruijn Graph compaction, genome assembly and metagenomic classification. Thus, in order to evaluate the various minimizer orderings, we implement a new distributed *k*-mer counting tool on Spark, called Discount. Discount can function as a pure *k*-mer counter, but can also double as a minimizer analysis tool, reporting detailed statistics about super-mers and *k*-mers in each bin. This allows us to freely evaluate various orderings on a realistic workload. Discount is freely available (on GitHub at https://github.com/jtnystrom/discount) and GPL licensed.

## 2 Materials and methods

In order to study minimizer orderings as well as their effect on practical tasks, we have implemented a distributed *k*-mer counting tool, Discount, on Apache Spark. Here, we briefly describe the design of this tool. Spark applications operate on *RDDs* (resilient distributed datasets), which are distributed collections of data, divided into some number of partitions. An application can execute some number of *stages* that operate on such partitions in parallel on a cluster. 

**Fig. 1.**
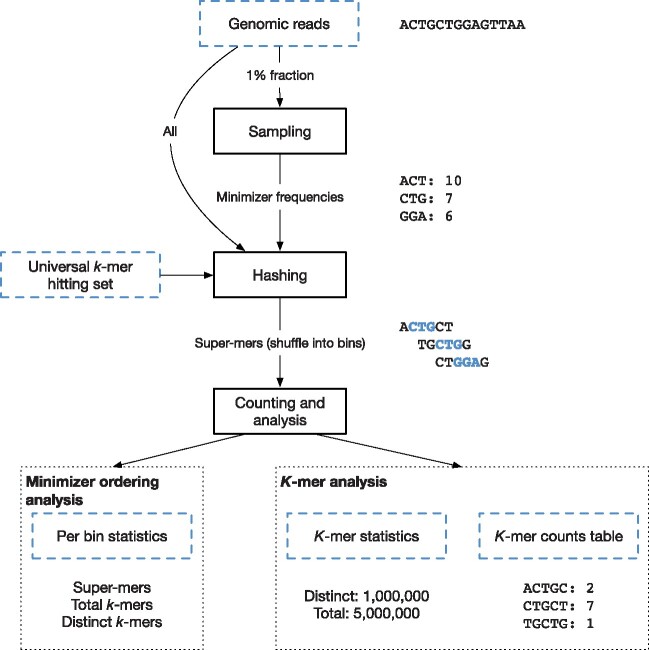
Internal stages of the Discount application. Here, we show a toy example with *m *=* *3 (minimizer length) and *k *=* *5 (*k*-mer length)

In order to read FASTA and FASTQ files efficiently into Spark, we use the FastDoop library (Ferraro Petrillo *et al.*, 2017). Next, the following stages are applied (Fig. 1).

- **Sampling (optional).** When sampling is used, a fraction (our default is 1%) of reads is sampled to obtain an estimate of minimizer frequencies in the data.
- **Hashing.** Genomic reads are split into super-mers. If desired, the minimizer frequency estimate from the previous stage, and optionally also a user-supplied universal hitting set, are used to configure the minimizer ordering. The resulting super-mers are encoded in the commonly used compact form of two bits per letter. Super-mers are shuffled into their assigned bins (i.e. by minimizer) such that each bin is located in its entirety in one partition, and thus on a single machine in a Spark cluster.
- **Processing.** In *k*-mer counting mode, in each bin super-mers are broken up into individual *k*-mers, sorted and counted. Optionally, a counts table or histogram is written to disk. Otherwise, only summary statistics is collected and aggregated, and then presented to the user. In minimizer analysis mode, a summary of the contents of bins is output in a table containing each bin’s number of super-mers, distinct *k*-mers and total *k*-mers. This allows for further downstream evaluation of the selected minimizer ordering’s behaviour and characteristics.

We use Discount to study the following minimizer orderings. As baselines for comparison, one may take the signature ordering, which is used by many tools in practice, and the naive random ordering.

**Signature.** We implement minimizer signatures according to the rules described in Deorowicz *et al.* (2015). See the introduction for details.

**Random.** A random ordering obtained by XORing each *m*-mer with a random constant. This ordering is different each time Discount runs.

**Frequency-sampled.** Here, we order minimizers from rare to common based on their estimated abundance in the actual dataset. For efficiency, we sample 1% of the data and use frequencies obtained from this fraction. Ties between equally frequent minimizers are resolved by ordering them lexicographically.

**Universal lexicographic.** We used the PASHA (Ekim *et al.*, 2020) tool to generate compact universal hitting sets for *k *=* *28, 55 and *m *=* *9, 10. For this ordering, we exclude those minimizers that are not in the universal set, and the included minimizers are ordered lexicographically (A < C < G < T). For example, for k=28,m=10, the set includes 1 67 178 *m*-mers and number of bins created would not be greater than this number. The sizes of our other generated sets are: 44 143 (k=28,m=9), 1 31 773 (k=55,m=10) and 34 719 (k=55,m=9).

**Universal random.** A random ordering on the universal sets used above, obtained by XORing each *m*-mer with a random constant. This ordering is different each time Discount runs.

**Universal frequency.** We propose a novel ordering, obtained by combining the frequency-sampled ordering and the universal set ordering. This ordering is established by sorting the universal sets used above according to the 1% sampled frequency count in the data. As above, ties between equally frequent minimizers are resolved lexicographically.

## 3 Results

We applied Discount to two short read NGS datasets: (i) part of a cow rumen metagenomic study (Hess *et al.*, 2011) (SRA run SRR094926), and (ii) part of the Tara Oceans marine metagenome study (Sunagawa *et al.*, 2015) (SRA run ERR599052). First, we used the first 100 million reads of both datasets (Table 1) to study the properties of the various orderings. As FASTQ files, each of these partial datasets is approximately 30 GB in size.

**Table 1.** Datasets

		Partial (100 million reads)		Full	
Dataset	k	Total	Distinct	Total	Distinct
Cow rumen	28	$7.39\times 10^{9}$	$5.09\times 10^{9}$	$7.23\times 10^{10}$	$2.94\times 10^{10}$
	55	$4.69\times 10^{9}$	$3.73\times 10^{9}$	$4.59\times 10^{10}$	$2.50\times 10^{10}$
Marine	28	$7.26\times 10^{9}$	$3.73\times 10^{9}$	$3.89\times 10^{10}$	$1.14\times 10^{10}$
	55	$4.57\times 10^{9}$	$3.04\times 10^{9}$	$2.44\times 10^{10}$	$1.04\times 10^{10}$

*Note*: Number of *k*-mers in the datasets used for minimizer ordering evaluation (partial) and *k*-mer counting (full) in this work.

**Fig. 2.**
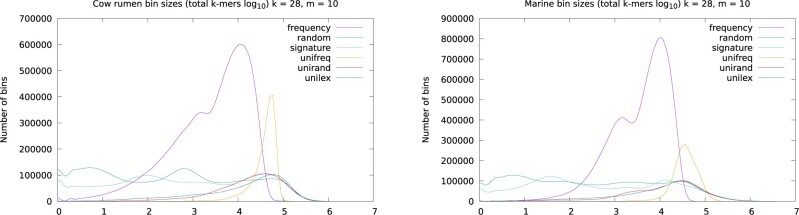
Density plots of *k*-mer bin distributions. Please see Table 2 for further details. Additional plots for *k *=* *55 are available in Supplementary Materials

We collected bin statistics for the various orderings (shown in Table 2). For each ordering, the total size of the largest 0.5% of bins gives an indication of the evenness of distribution (the smaller, the better). The signature ordering resulted in an uneven distribution, but produced long super-mers. The universal lexicographic and universal random orderings produced even longer super-mers, and were slightly more evenly distributed. The frequency ordering greatly improved evenness, but super-mers were much shorter. Finally, the universal frequency ordering produced the best max/mean ratio, the best evenness, and also almost as long super-mers as the signature ordering. Figure 2 shows density plots of the bin size distributions for the two datasets. The frequency ordering had a larger area under the curve since it generated a much larger number of mostly very small bins. Universal frequency bin sizes were mainly concentrated in a single peak.

**Table 2.** Minimizer ordering measurements

				Bin sizes (total k-mers)					
k	m	Ordering	Bins	Mean	Max	Max/mean	Std.dev	Avg s.mer	Top 0.5%
**Cow rumen metagenome dataset**									
28	10	random	4 02 181	18 373.98	14 08 363	76.65	44 412.59	9.28	11.66%
		frequency	9 85 599	7 497.64	2 02 535	27.01	8 965.39	5.75	3.25%
		signature	4 15 899	17 767.94	13 52 062	76.10	40 958.18	9.02	8.81%
		universal lex	1 66 577	44 361.86	18 93 021	42.67	59 794.00	9.84	4.87%
		universal rand	1 67 115	44 219.05	45 30 489	102.46	61 420.43	9.42	5.21%
		universal freq	1 67 172	44 203.97	3 94 768	8.93	18 921.38	9.27	1.14%
55	10	random	2 59 619	18 070.26	18 71 228	103.55	55 389.36	16.15	13.04%
		frequency	8 89 414	5 274.69	2 87 304	54.47	8 857.77	11.31	5.01%
		signature	2 09 023	22 444.34	18 29 255	81.50	53 684.77	16.13	9.28%
		universal lex	1 23 308	38 046.05	30 67 656	80.63	69 920.79	16.28	7.29%
		universal rand	1 25 272	37 449.57	42 76 472	114.19	71 767.70	16.16	7.68%
		universal freq	1 31 704	35 620.66	3 12 687	8.78	23 950.62	15.97	1.51%
**Marine metagenome dataset**									
28	10	random	5 36 419	13 534.92	74 08 859	547.39	69 815.50	7.64	19.95%
		frequency	1 002 163	7 244.72	82 931	11.45	7173.75	5.12	2.52%
		signature	415 718	17 464.70	51 79 699	296.58	68 689.92	9.20	19.92%
		universal lex	1 66 520	43 600.70	88 15 602	202.19	1 19 830.20	9.78	13.74%
		universal rand	1 66 895	43 502.73	50 85 446	116.90	97 268.02	9.45	11.86%
		universal freq	1 67 173	43 430.39	5 35 913	12.34	26 234.79	9.00	2.02%
55	10	random	2 08 541	21 896.81	48 26 566	220.42	78 406.11	15.88	17.23%
		frequency	9 93 329	4597.05	68 616	14.93	6635.20	10.41	4.25%
		signature	1 96 514	23 236.93	55 94 282	240.75	83 778.67	16.05	18.17%
		universal lex	1 23 364	37 015.51	1 42 23 589	384.26	1 42 466.09	16.03	18.92%
		universal rand	1 23 921	36 849.14	59 36 297	161.10	98 391.61	16.32	15.54%
		universal freq	1 31 765	34 655.50	1 02 733	2.96	15 219.88	15.62	1.16%

*Note*: Results obtained when using Discount to break up each dataset into binned super-mers. Here, *m* represents the minimizer length, so that for an unconstrained ordering, there would be a theoretical maximum of $4^{m}$ different minimizers (bins). Bin sizes are measured as the total number of *k*-mers in each bin prior to counting distinct items (i.e. as the sum of super-mer lengths in that bin). The average super-mer length is measured as a number of *k*-mers. The rightmost column gives the proportion of *k*-mers in the largest 0.5% of bins. Data for *m *=* *9 may be found in Supplementary Materials.

Since they depend on a random constant, the random ordering and the universal random ordering were in practice different specific orderings for each run of Discount. The average super-mer length for the random ordering on the marine dataset (*k *=* *28) is an outlier (7.64) and shows the volatility of this ordering.

As for the sampling fraction for the frequency-sampled orderings, in practice we have found that 1% of reads produced consistent results, and can be sampled quickly. Increasing the fraction to 10% had only very minor effects in terms of the metrics we study here.

Next, we compared the performance of *k*-mer counting on Discount with FastKmer (Ferraro Petrillo *et al.*, 2019). FastKmer is a highly efficient distributed *k*-mer counting tool based on Spark, which uses a variant of the signature ordering. For this comparison, we applied the two tools to the full data from run SRR094926, cow rumen metagenomics data (see above), from which we previously used only 100 million reads (Table 1). The size of this dataset is about 314 GB as uncompressed FASTQ files.

We ran the benchmarks on the Google Cloud Platform (GCP) using three different configurations (Table 3). In each case, four worker machines with sixteen cores each were used, and the cluster master machine was an n1-standard-4 machine with four CPUs. All machines were from the Google Cloud N1 series, with Intel Xeon CPUs running at 2.7–3.2 GHz (all-core turbo frequency). The version of Apache Spark used was 2.4.6. For FastKmer, we used four n1-highmem-16 machines, for a total of 64 CPUs and 256 GB executor RAM (since FastKmer would not run with less memory). The FastKmer authors’ recommended best settings from Ferraro Petrillo *et al.* (2019) were used: x=3,b=8192. However, we increased parallelism (partitions) from the recommended 320 to 2000, since this gave better performance in our setting. For FastKmer, the number of bins was limited to 8192 as recommended. We also tried larger numbers but did not see a performance improvement. For Discount, the universal frequency ordering was used with the same universal sets as in the previous section, *m *=* *10. The number of bins was not constrained and most likely exceeded the number shown in Table 2 (but would not have been larger than 1 67 178, the size of the universal set).

**Table 3.** Resource configurations used for performance measurements

Benchmark	Worker machine type	Workers	Cores	RAM (GB)	Executor RAM (GB)	Partitions (parallelism)
FastKmer	n1-highmem-16	4	64	416	256	2000
Discount high memory	n1-standard-16	4	64	240	87	4000
Discount low memory	n1-highcpu-16	4	64	57.6	34	14 000

*Note*: Cores and memory (RAM) are reported as totals for all worker machines.

The two applications were performing very similar tasks: outputting a final table with counts for each *k*-mer. Generating this table involves allocating a large amount of strings and writing the data to disk. Hence, this benchmark shows the overall performance effects of non-trivial processing of *k*-mer bins. The outputs from the two applications were identical, except that FastKmer unified *k*-mers with their reverse complements, unlike Discount.

In a Spark cluster, not all the memory available on the machines is assigned to Spark executors (which run the actual tasks), since some memory is reserved for the operating system, task management and other functions. For the Discount high memory case, we limited executor memory to test the efficiency of our method. Thus, the total executor memory in that case was only 87 GB across all four machines. For the Discount low memory case, we increased the number of Spark partitions, making them smaller to further limit memory pressure. We also reduced the maximum MapReduce split size (for the underlying file inputs) from the default 128–64 MB.

We measured the time required by running Discount as well as FastKmer on the full dataset (Table 4). Since FastKmer is internally divided into two main stages, we break down its runtime in the same way as we do for Discount. However, the precise algorithms used by these stages are different between the two applications. To test scaling to a larger number of worker machines, we also ran the Discount high memory case on sixteen worker machines with four CPUs each. Performance did not change significantly from the four machine case.

**Table 4.** Performance comparison

			Runtime (min)				GB
Case	k	m	Sample	Hash	Process	Total	Shuffle
FastKmer	28	10		21	57	78	125.4
	55	10		18	43	60	73.1
Discount high memory	28	10	1.4	12	43	57	159.4
	55	10	1.4	8.9	39	51	89.5
Discount low memory	28	10	2.0	16	60	79	172.9
	55	10	1.7	11	49	63	100.3

*Note*: Cow rumen full dataset. For Discount, the universal frequency ordering was used. FastKmer does not have a sampling stage, so no timing is reported. The shuffle data, which is stored on disk when not needed in memory, corresponds to all generated super-mers, binned and partitioned. These are shuffled across the network to the correct machine between the hashing stage and the processing stage. The size, which is sensitive to super-mer length and to the number of partitions, is a total across all of the machines.

We also measured, for the cow rumen dataset and *k *=* *28, the memory usage of each minimizer ordering, to be able to separate their performance benefits from other factors (Table 5). When the memory pressure of Java VM applications, such as Discount, increases, the application spends a higher percentage of its CPU time in garbage collection (GC). We adjusted the total heap size in 16 GB increments from a baseline to find the smallest size that would allow Discount to spend at most 15% of its time in GC. The worst performing orderings in this comparison, universal lexicographic and universal random, were already relatively frugal. The lowest memory usage was achieved by the frequency ordering (71 GB), with universal frequency a close second (87 GB).

**Table 5.** Memory usage of different orderings

			Time (min)			GB	
Ordering	k	m	Hash	Process	Total	Shuffle	Memory
Frequency	28	10	19	39	60	259.7	71
Frequency	28	9	14	44	60	219.9	135
Random	28	10	14	42	57	165.7	119
Signature	28	10	13	43	57	163.9	135
Universal lex	28	10	12	44	57	151.4	151
Universal rand	28	10	12	43	57	150.2	151
Universal freq	28	10	12	43	57	159.4	87

*Note*: Cow rumen full dataset. Executor memory required to run with less than 15% of CPU time spent in garbage collection. Other settings were the same as in the *Discount high memory* case.

Finally, we compared the performance of Discount with the traditional, non-distributed *k*-mer counters KMC3 and Jellyfish to evaluate the benefits of distributed processing. For KMC3 and Jellyfish we used a single machine with the same total resources as total of the four Discount worker machines—240 GB RAM and 64 cores—to examine the effect of distributing the workload (Table 6). The machine had a single 4 TB HDD disk, and the Discount workers had one 1 TB disk each. As before, we measured the task of generating a full table with the counts of all *k*-mers. The total time required was about twice as long for KMC3 (114 min versus 57) and more than four times as long for Jellyfish (252 min). However, the benefit is task dependent: when only generating summary statistics for a dataset, we found that KMC3 was faster than Discount. Full details of the commands used are given in the supplementary materials.

**Table 6.** Comparison with non-distributed k-mer counters

		Runtime (min)			GB
Case	k	Count	Dump	Total	Temp data
Jellyfish	28	124	128	252	302
KMC3	28	28	86	114	165
Discount	28			57	159

*Note*: Cow rumen full dataset. The total size of the generated *k*-mer counts table was 851 GB. For comparison, we reproduce the Discount high memory case from Table 4. Its stages are not directly comparable with the count and dump operations.

## 4 Discussion

In this work, first, we compared six minimizer orderings when applied to two partial metagenomics datasets: random, signature, frequency, universal lexicographic, universal random and universal frequency. The signature ordering has been a practical choice for many tools since it yields relatively long super-mers and avoids certain large bins. However, it produces a high 0.5% bin fraction: up to 19.92% for the marine dataset for k=28,m=10.

The universal lexicographic and universal random orderings yielded even longer super-mers. However, they were only slightly more evenly distributed. The frequency-sampled ordering dramatically improved evenness of distribution, as well as the size of the maximum bin, but at the expense of a much larger number of bins for a given value of *m*. As Figure 2 shows, this ordering yielded a large amount of small bins. For some applications, having to maintain so many very small bins will lead to undesirable overhead. Moreover, super-mers are very short, meaning that the bins cannot be stored efficiently in this form.

The universal frequency ordering consistently obtained the best evenness of distribution, the lowest max/mean ratio, and also restored the long super-mers. For example, for the marine dataset, k=55,m=10, for a bin number comparable to the universal lexicographic ordering, the 0.5% bin fraction was reduced from 18.92% to 1.16%. Super-mers were almost as long as for the signature and universal cases. Thus, for a given number of desired bins, this ordering provided the best balance of long super-mers and an even distribution.

Next, we applied the universal frequency ordering to distributed *k*-mer counting, to test a practical application. We evaluated a high memory as well as a low memory scenario while comparing against FastKmer, an existing similar *k*-mer counter. For the former, although the amount of executor memory assigned to Discount was only 34% of what was assigned to FastKmer, Discount ran faster. We believe that this reflects various costs of processing larger bins. For example, both FastKmer and Discount have to sort all *k*-mers in each bin as part of counting, and the cost of sorting increases more than linearly as the array to be sorted grows longer. For the low memory scenario, we carefully tuned Spark to test the limits of our approach. Discount was running on a low memory machine type, with a total of 64 CPUs and only 34 GB total executor memory, around 1/8 of the FastKmer memory. Even with this minimal resource allocation, Discount ran at nearly the same speed as FastKmer.

We also evaluated the effects on runtime and memory usage of each minimizer ordering for *k *=* *28 on the cow rumen dataset. Generally, orderings that produced a greater number of smaller bins required less memory than the ones producing fewer and larger bins. A notable exception was the universal frequency ordering, which had the second lowest memory usage, despite generating a number of bins similar to those yielded by the universal lexicographic and universal random orderings. We include the frequency ordering for *m *=* *9 to show the effect of this parameter. For this ordering, increasing *m* to 10 produced around four times more bins (2 52 033 versus 9 85 599, details in Supplementary Materials). This by itself reduced the memory requirement substantially.

Although the frequency ordering had the lowest memory usage, this is largely because it was able to generate around six times more bins than the universal frequency ordering (Table 2), which is second lowest. It also does this while generating much shorter super-mers, which inflates the size of the shuffle data. If this additional memory usage reduction is desired, universal frequency for *m *=* *11 may be a better choice than frequency for *m *=* *10.

Finally, we compared the performance of Discount with the non-distributed *k*-mer counters KMC3 and Jellyfish. Since the task was to generate a full *k*-mer counts table, which is more than 800 GB in size, disk I/O became a limiting factor, and Discount thus benefited from having access to four disks operating independently on separate machines, as well as from inputs and outputs being stored on a distributed filesystem in the cloud. A full investigation of the performance of Discount on large clusters is beyond the scope of this work. However, since a typical single machine can only support a small number of disks, but Spark clusters can have thousands of worker nodes, Discount should be an attractive choice for very large datasets.

Distributed *k*-mer counters can be divided into two categories: out-of-core, (which keep some data on disk) and in-core methods (which keep all data in memory). FastKmer and Discount are both in the former category, since Spark relies on the ability to spill data to disk when necessary. Given the comparison between FastKmer and other tools such as KCH, ADAM and BioPig in Ferraro Petrillo *et al.* (2019), which are also out-of-core, this would make Discount both the fastest and the most memory efficient distributed *k*-mer counter in this category. On machines with a given amount of memory, the maximum amount of data that Discount can analyse should be much larger than for comparable existing tools.

In the present work, we have only studied selected minimizer orderings of interest, and we leave a broader comparison with other binning methods for future work. For example, Efe (2018) suggests a method based on sums of integers associated with the letters of a *k*-mer.

Many *k*-mer processing tools unify each *k*-mer with its reverse complement, treating them as the same value. This is made possible in part by restricting minimizers and super-mers in certain ways. Unfortunately, with our minimizer ordering this kind of optimization is not currently possible. This has been recognised as an open problem for universal sets (Marçais *et al.*, 2017). In general, research in universal *k*-mer sets is currently ongoing (DeBlasio *et al.*, 2019; Zheng *et al.*, 2020), and future results may further improve the universal frequency ordering.

## 5 Conclusion

In this work, we have investigated the formation of binned super-mers from genomic sequences by using minimizers, a common technique in omics data analysis tools. To support the investigation, we implemented a new distributed *k*-mer counting tool, Discount, which also has minimizer ordering analysis functionality. We sought to achieve an even distribution of bin sizes, aiming for improvements such as memory usage reduction, efficient storage on disk and increasing the overall processing speed. By combining frequency-sampled minimizers with universal *k*-mer sets, we obtained the universal frequency ordering. To the best of our knowledge, the present work is the first time this combined ordering has been used. Relative to minimizer signatures, the fraction of *k*-mers stored in the largest 0.5% of bins was reduced by as much as from 18.17% to 1.16% (for *m *=* *10, *k *=* *55, marine dataset) while still yielding long super-mers. Furthermore, the cost of sampling is small: for the full dataset, only around 5% of the runtime was spent sampling 1% of the reads. Using Discount, compared with the fastest existing out-of-core distributed *k*-mer counting tool, we were able to count *k*-mers in a metagenomic dataset at comparable speed using only 14% of the memory. Considering these benefits, we believe that frequency-sampled universal minimizers would significantly improve the performance of many tools that use minimizers to construct binned super-mers, and that this should be a preferred strategy for producing evenly sized bins. With this minimizer ordering, Discount expands the practical boundaries of analysis of very large omics datasets.

## Financial Support

none declared.

*Conflict of Interest:* none declared.

## Supplementary Material

btab156_Supplementary_Data
