# Compact and evenly distributed *k*-mer binning for genomic sequences

**DOI:** 10.1093/bioinformatics/btab323

**Published:** 2021-05-10

**Authors:** Johan Nyström-Persson, Gabriel Keeble-Gagnère, Niamat Zawad

*Bioinformatics* (2021) doi: 10.1093/bioinformatics/btab156

In the originally published version of this manuscript, the layout of Tables 1, 2 and 4 was incorrect thus affecting the readability of the data. This has now been corrected online.

The Publisher apologizes for these errors.

