# Supplementary material for: Compact and evenly distributed k-mer binning for genomic sequences
Source: Bioinformatics. 2021 Mar 8;37(17):2563–9. doi: 10.1093/bioinformatics/btab156 (PMC8428581; doi:10.1093/bioinformatics/btab156)
Supplement: btab156_Supplementary_Data [file btab156_supplementary_data.pdf]

Compact and Evenly Distributed k-mer Binning for Genomic Sequences

Supplementary materials

Johan Nyström-Persson, Gabriel Keeble-Gagnère, and Niamat Zawad

February 2021

Bin statistics generated by various minimizer orderings

Minimizer orderings

- **Random** A random ordering of all m-mers.
- **Frequency** A sampled (1%) frequency ordering of all m-mers, from rare to common.
- **Signature** The minimizer signature ordering, as implemented by KMC2. Gives lower priority to minimizers starting with AAA or ACA, or containing AA anywhere, except for AA at the start.
- **Universal lexicographic** A lexicographic ordering of a compact universal hitting set.
- **Universal random** A random ordering of a compact universal hitting set.
- **Universal frequency** A sampled (1%) frequency ordering of a compact universal hitting set, from rare to common.

In all of the orderings, any ties between items of equal priority were resolved lexicographically.

Universal sets

To generate universal set orderings, compact universal hitting sets generated by the PASHA algorithm were used. These sets have the following sizes.

k	m	m-mers
28	10	167,178
28	9	44,143
55	10	131,773
55	9	34,719

Bin statistics (cow rumen)

The following measurements were obtained using the first 100,000,000 reads from the cow rumen dataset (SRA accession SRR094926). This dataset has the following properties.

k	Total k-mers	Distinct k-mers
28	7,389,666,230	5,090,549,289
55	4,691,382,750	3,728,398,897

Throughout this document, bin sizes are measured as the total number of k-mers, including duplicates. Super-mer lengths are measured as the number of overlapping k-mers. For example, a super-mer of length 5 when $k = 28$ would be a sequence of length 32. *Top 0.5%* gives the total size of the largest 0.5% of the bins.

We also reproduce the values for $m = 10$ here for convenience.

				Bin sizes (total k-mers)					
**k**	**m**	**Ordering**	**Bins**	**Mean**	**Max**	**Max/ mean**	**Std.dev**	**Avg s.mer**	**Top 0.5%**
28	10	random	402,181	18,373.98	1,408,363	76.65	44,412.59	9.28	11.66%
		frequency	985,599	7497.64	202,535	27.01	8965.39	5.75	3.25%
		signature	415,899	17,767.94	1,352,062	76.10	40,958.18	9.02	8.81%
		universal lex	166,577	44,361.86	1,893,021	42.67	59,794.00	9.84	4.87%
		universal rand	167,115	44,219.05	4,530,489	102.46	61,420.43	9.42	5.21%
		universal freq	167,172	44,203.97	394,768	8.93	18,921.38	9.27	1.14%
	9	random	139,680	52,904.25	4,984,198	94.21	144,962.35	8.99	9.73%
		frequency	252,033	29,320.23	224,259	7.65	34,824.45	6.02	2.76%
		signature	118,073	62,585.57	3,430,989	54.82	152,146.48	9.43	9.07%
		universal lex	44,013	167,897.35	4,047,350	24.11	222,499.52	10.05	4.70%
		universal rand	44,115	167,509.15	6,143,109	36.67	236,775.75	10.11	4.89%
		universal freq	44,141	167,410.49	513,956	3.07	73,576.53	9.61	0.95%
55	10	random	259,619	18,070.26	1,871,228	103.55	55,389.36	16.15	13.04%
		frequency	889,414	5274.69	287,304	54.47	8857.77	11.31	5.01%
		signature	209,023	22,444.34	1,829,255	81.50	53,684.77	16.13	9.28%
		universal lex	123,308	38,046.05	3,067,656	80.63	69,920.79	16.28	7.29%
		universal rand	125,272	37,449.57	4,276,472	114.19	71,767.70	16.16	7.68%
		universal freq	131,704	35,620.66	312,687	8.78	23,950.62	15.97	1.51%
	9	random	69,966	67,052.32	4,318,856	64.41	180,145.77	16.38	12.17%
		frequency	231,139	20,296.80	321,958	15.86	33,879.72	11.53	4.35%
		signature	64,369	72,882.64	4,928,318	67.62	190,408.32	16.33	10.07%
		universal lex	33,070	141,862.19	7,310,195	51.53	260,641.17	16.42	7.24%
		universal rand	33,190	141,349.28	3,975,344	28.12	250,657.48	16.70	6.83%
		universal freq	34,708	135,167.19	397,257	2.94	98,179.22	16.24	1.27%

Bin statistics (marine metagenome)

The following measurements were obtained using the first 100,000,000 reads from the marine metagenome dataset (SRA accession ERR599052). This dataset has the following properties.

k	Total k-mers	Distinct k-mers
28	7,260,388,843	3,726,243,999
55	4,566,381,859	3,042,228,761

				Bin sizes (total k-mers)					
**k**	**m**	**Ordering**	**Bins**	**Mean**	**Max**	**Max/ mean**	**Std.dev**	**Avg s.mer**	**Top 0.5%**
28	10	random	536,419	13,534.92	7,408,859	547.39	69,815.50	7.64	19.95%
		frequency	1,002,163	7244.72	82,931	11.45	7173.75	5.12	2.52%
		signature	415,718	17,464.70	5,179,699	296.58	68,689.92	9.20	19.92%
		universal lex	166,520	43,600.70	8,815,602	202.19	119,830.20	9.78	13.74%
		universal rand	166,895	43,502.73	5,085,446	116.90	97,268.02	9.45	11.86%
		universal freq	167,173	43,430.39	535,913	12.34	26,234.79	9.00	2.02%
	9	random	140,972	51,502.35	4,692,415	91.11	148,977.06	8.91	12.31%
		frequency	256,774	28,275.40	190,077	6.72	26,050.40	5.32	2.16%
		signature	115,021	63,122.29	10,974,813	173.87	238,833.59	9.61	18.95%
		universal lex	44,002	165,001.34	19,499,484	118.18	427,240.83	9.95	12.97%
		universal rand	44,093	164,660.80	11,659,078	70.81	324,481.79	9.90	10.34%
		universal freq	44,143	164,474.30	692,028	4.21	72,388.41	9.32	1.27%
55	10	random	208,541	21,896.81	4,826,566	220.42	78,406.11	15.88	17.23%
		frequency	993,329	4597.05	68,616	14.93	6635.20	10.41	4.25%
		signature	196,514	23,236.93	5,594,282	240.75	83,778.67	16.05	18.17%
		universal lex	123,364	37,015.51	14,223,589	384.26	142,466.09	16.03	18.92%
		universal rand	123,921	36,849.14	5,936,297	161.10	98,391.68	16.32	15.54%
		universal freq	131,765	34,655.50	102,733	2.96	15,219.88	15.62	1.16%
	9	random	78,070	58,490.87	5,876,699	100.47	151,623.28	16.17	22.82%
		frequency	255,909	17,843.77	175,818	9.85	24,663.22	10.53	3.72%
		signature	62,662	72,873.22	12,703,061	174.32	270,944.32	16.24	18.53%
		universal lex	33,058	138,132.43	29,922,492	216.62	502,301.46	16.14	18.05%
		universal rand	33,294	137,153.30	8,125,032	59.24	327,469.10	16.48	9.98%
		universal freq	34,715	131,539.16	305,236	2.32	56,089.27	15.86	1.02%

Density plots of bin distributions

A kernel density estimate, using Gaussian kernels, has been used to generate these density plots from bins generated from the two 100,000,000 read datasets above. Bin sizes are measured as the total number of k-mers, including duplicates.

For convenience, we also reproduce the plots for $k = 28$ here, although they have already been given in the main paper.

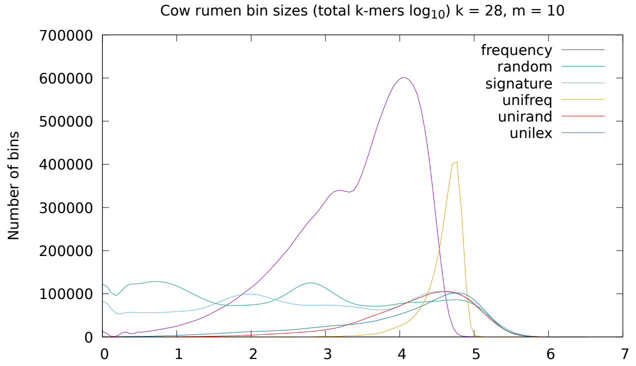

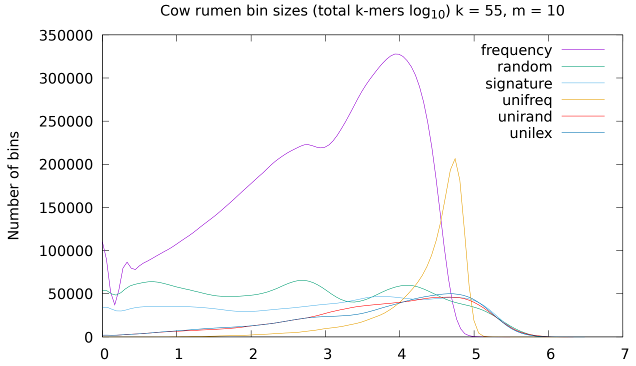

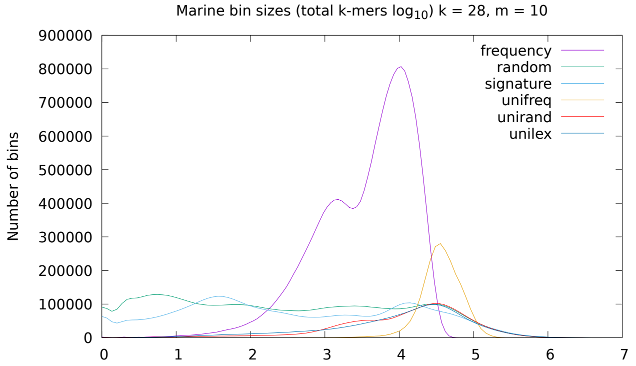

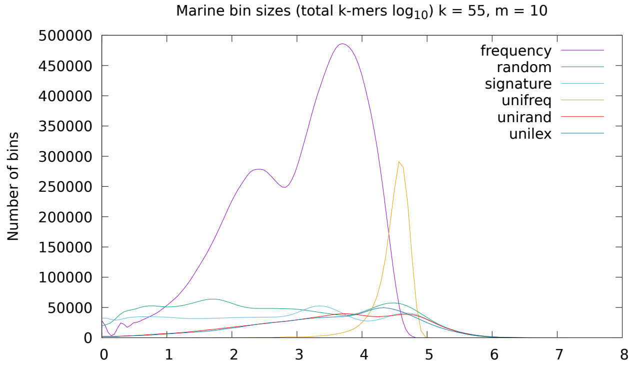

Commands used to invoke KMC3 and Jellyfish

Here we give the commands used when comparing the traditional k-mer counters KMC3 and Jellyfish with Discount.

A single machine with 64 CPUs, a single 4 TB HDD (standard persistent) disk, and 240 GB RAM was used. The machine was from the Google Cloud N1 series with Intel Xeon CPUs running at 2.7 - 3.2 GHz (all-core turbo frequency).

The memory limit passed to the tools was 220 GB, to allow the rest to be used as disk cache by the operating system. Parameters were tuned to optimise speed while producing outputs as similar as possible to those from Discount. Inputs were uncompressed.

Discount was writing the k-mer counts output table as 4,000 separate files on the Google Cloud distributed filesystem (equivalent to the number of partitions used in Spark). When generating equivalent data, Jellyfish and KMC3 output a single large file.

Jellyfish

Jellyfish version 2.2.10 was used. The following command was used to determine the initial hash size:

```
jellyfish mem -m 28 --mem=$((220 * 1024 * 1024 * 1024))
```

The number obtained (34359738368) was passed to the `-s` argument in the following command.

Command for step 1:

```
jellyfish count -F 2 -t 64 -L 1 -m 28 -s 34359738368 file1.fasta file2.fasta
```

Command for step 2:

```
jellyfish dump -L 1 -t -c -o output.tsv mer_counts.jf
```

KMC3

KMC3 version 3.0.0 was used.

Command for step 1:

```
kmc -k28 -sm -m220 -t64 -b -ci1 -fa @inputs.txt kmc_out /tmp
```

Command for step 2:

```
kmc_dump -ci1 kmc_out output.tsv
```
